# Surgical patients’ experiences with the Patients’ Safety Checklist (PASC): a qualitative interview study

**DOI:** 10.1136/bmjopen-2025-105554

**Published:** 2025-10-05

**Authors:** Kristin Harris, Hilde Valen Wæhle, Anette Storesund, Stig Harthug, Randi Julie Tangvik, Denisa Monsen Lukcova, Wenche Håvik, Åse Humberset, Evelyn Stavang, Kjetil Hagerup, Åshild Teigland Tepstad, Ann Kristin Sandsbakk Austarheim, Andy Healey, Nick Sevdalis, Arvid Steinar Haugen

**Affiliations:** 1 Department of Anaesthesia and Intensive Care, Haukeland University Hospital, Bergen, Norway; 2 Department of Health and Caring Sciences, Western Norway University of Applied Sciences, Bergen, Norway; 3 Department of Global Public Health and Primary Care, University of Bergen, Bergen, Norway; 4 Department of Research and Development, Haukeland University Hospital, Bergen, Norway; 5 Department of Clinical Medicine, University of Bergen, Bergen, Norway; 6 Department of Breast and Endocrine Surgery, Haukeland University Hospital, Bergen, Norway; 7 Department of Heart Disease, Section of Cardiothoracic Surgery, Haukeland University Hospital, Bergen, Norway; 8 Department of Neurosurgery, Haukeland University Hospital, Bergen, Norway; 9 Department of Surgery, Helse Førde HF, Førde, Norway; 10 Helsetorget GP Practice, Bergen, Norway; 11 Department of Medicine, Section of Clinical Nutrition, Haukeland University Hospital, Bergen, Norway; 12 Department of Nursing and Health Promotion Acute and Critical Illness, Faculty of Health Sciences, Oslo Metropolitan University, Oslo, Norway; 13 King's Health Economics, Health Service, and Population Research Department, King’s College London, London, UK; 14 Department of Medicine, National University Hospital, Singapore

**Keywords:** SURGERY, Safety, Patient Participation

## Abstract

**Background:**

Engaging patients in surgical safety is challenging and has not been thoroughly investigated. Although surgical checklists and other safety protocols have been introduced across various surgical fields, preventable adverse events still occur, highlighting the need for additional research. A Patient’s Safety Checklist (PASC) has been developed and validated for use by surgical patients. Its effect on patient safety and patient outcomes is currently being investigated in a Stepped Wedge Cluster Randomised Controlled Trial (NCT03105713). In connection with this trial, we have examined elective patients’ experiences with using the PASC.

**Methods:**

An exploratory qualitative study was conducted based on individual in-depth telephone interviews with 31 elective surgical patients. The interviews were carried out across three Norwegian hospitals including seven surgical specialties. The patients interviewed were part of the trial’s intervention arm and had used PASC. The interviews were transcribed verbatim, and reflective thematic analysis was applied.

**Result:**

Three themes were identified in the data: patient awareness, patient actions and utility value. Patients perceived PASC to increase awareness around surgical information, preparations, what to speak up about and which information to seek and repeat. This awareness led to a series of actions, such as ensuring medication control, optimising their own health, contacting healthcare professionals, asking questions, and for some no actions were needed. Patients perceived PASC to have high utility value for their surgical preparation.

**Conclusions:**

The PASC enhanced patients’ involvement in surgical care and safety by ensuring they received systematic, accurate, clear, and understandable information and instructions throughout the surgical pathway. It is one of the few existing interventions that specifically focuses on assisting patients in preparing for surgery and managing their recovery. Further research is needed on the implementation of PASC and its adaptation to other clinical settings.

**Trial registration number:**

NCT03105713.

**STRENGTHS AND LIMITATIONS OF THIS STUDY**

- Exploratory qualitative study utilising reflective thematic analysis.
- Part of a larger Stepped Wedge Cluster Randomised Controlled Trial.
- The study includes a strategically sampled variety of surgical patients across seven surgical specialties and three hospitals.
- Participants’ recall bias may have occurred.
- Patients’ Safety Checklist has only been developed and studied in Norwegian settings.

## Background

 Patient involvement in surgical safety is a complex issue that remains underexplored.^1^ Despite the implementation of surgical safety checklists and other safety measures across surgical specialties,^2 3^ preventable adverse events (eg, medication errors and healthcare-associated infections) continue to occur and further research is warranted.^4^ The WHO’s current action plan aims to eliminate all preventable harm by 2030.^5^ To achieve this goal, WHO emphasises that patients’ involvement must be targeted at all levels of care within healthcare organisations.^5 6^ To date, few checklists have been developed for patients to use in surgical care, and those that do exist are mostly tailored to specific surgical procedures rather than being generally applicable.^7^,^10^ Early evidence indicates that such patient-driven checklists may reduce hospital length of stay (LOS) and readmissions.^7^,^9^

This paper focus on a surgical Patient’s Safety Checklist (PASC) intended for general application across any perioperative care pathway, which our group has been working on over several years. PASC has been co-developed with patients, healthcare professionals, managers and safety researchers, and its content has been validated by surgical patients.^11 12^ The checklist is intended for use by patients, with the ultimate aim to empower surgical patients to actively participate in their own safety throughout their surgical pathway (from surgical preparation to recovery).^11 12^ It is a generic checklist tailored for most types of surgery. It is also customisable, allowing items to be added or removed to match the specific information needs and workflow of each surgical specialty.^11^ PASC is divided into two parts: one to be completed before surgery and one before discharge.^11^ Both parts of PASC are distributed electronically and on paper to elective surgical patients between one and 8 weeks before surgery, depending on the severity of their condition and the urgency of the elective surgery.^11^ The first part consists of 26 core and three advisory items, divided into three sections. The first section addresses health optimisation, including medication use, dental health, existing medical conditions, lifestyle and self-nutritional status screening.^11 13^ The second section covers patients’ presurgical and postsurgical preparations and information to be considered 2 weeks before surgery. The third section addresses presurgical routines such as showering, fasting, allergies and marking of the surgical site. The second part of PASC is designed to help patients seek and clarify information before their discharge from the hospital. This part contains 22 items, which cover potential complications, activities and restrictions, medications, pain relief, gastrointestinal functions and further plans and follow-up.^11^

There is currently a knowledge gap regarding how a checklist tool like PASC may influence patients’ involvement and perceived safety.^10^ Hence, the aim of this study was to explore surgical patients’ experiences of using PASC throughout their surgical pathway.

## Methods

This was an exploratory qualitative study, based on in-depth individual patient interviews. The study was part of a Stepped Wedge Cluster Randomised Controlled Trial (SWCRCT),^14^ designed to investigate the impact of PASC on elective surgical complications, LOS in hospital and mortality, as well as patients’ experiences of using PASC. Data in this trial were collected from November 2021 to February 2024. The elective surgical patients in the control group received care as usual, while the patients in the intervention group received PASC both electronically and by mail, 1–8 weeks before surgery.^14^ In this part of the study, participants from the intervention arm were asked to share their experiences of using PASC throughout their surgical pathway. The Consolidated criteria for Reporting Qualitative research (online supplemental 1) and the Reflexive Thematic Analysis Reporting Guidelines were followed during the study to ensure transparency.^15 16^

### Patient and public involvement

Since the inception of the PASC project in 2012, patient representatives from the Western Norway Health Trust have played an active and integral role. The concept of a patient-driven surgical checklist originated from these representatives, who, along with surgical patients, contributed significantly to the design, development, validation and feasibility testing of PASC.^11 12 17^ In this qualitative study, their primary involvement was cocreation of the interview guide and reviewing the results.

### Settings and participants

The study was carried out across three Norwegian hospitals in the Western Norway health region, with 34 000 surgical operations being performed annually. Seven surgical specialties across eight surgical wards participated in the SWCRCT, from which patients were recruited by healthcare professionals at each ward. This included the following surgical wards: ear, neck, throat (ENT)/maxillofacial; cardiac-thoracic; neuro; breast and endocrine; gastrointestinal; general surgery, and two orthopaedic wards.^14^ Patients who were eligible to participate were elective surgical patients ≥18 years, without cognitive impairment, fluent in Norwegian and part of the SWCRCT intervention arm using the PASC intervention.^14^ The criterion for cognitive healthy impairment was determined and assessed by and in collaboration with the ward nurses in each cluster. Patients enrolled in the trial were strategically sampled to participate in in-depth interviews postdischarge. They were invited either before discharge from the hospitals or contacted after discharge via telephone by ASH, KHar, HVW and RJT. If they accepted the invitation, an interview appointment was scheduled. No reminders were sent out. ASH, KHar and HVW carried out the phone interviews from a hospital office phone. No participants withdrew from this study. A total of 31 patients were interviewed approximately 1–5 weeks after surgery.

### Data collection

The telephone interviews were performed over a period of 3 months, from November 2023 to mid-January 2024. A semistructured interview guide was developed based on previous PASC studies.^11^ There were no prior relationships between the participants and the researchers. The interview guide explored patients’ perceived experiences of using PASC. Participants were initially asked to share openly their overall experience, including both positive and negative aspects, which resulted in detailed narratives of their experiences of using PASC. Additionally, they were prompted to elaborate on specific PASC item topics such as surgical preparations, information needs and discharge preparations. Finally, they were asked whether PASC provided them with a sense of safety or not, and if they would consider using it again (online supplemental 2). The interview guide was piloted with three patients (included in the total n) and only minor language adjustments were made. The interviews lasted from eight to 40 min (mean duration=15.5 min), were digitally recorded, transcribed verbatim and quality ensured up against the recordings by ASH, KHar and HVW. Data information power was considered before the data collection. In addition, this was discussed and agreed on by ASH, KHar and HVW before analysis was carried out, as recommended by Braun and Clarke.^18^

### Data analysis

Reflective thematic analysis with a semantic approach were used to analyse the data.^16^ In a semantic approach, themes are derived from the explicit or surface-level meanings within the data, with the analyst focusing solely on what participants have said, without seeking interpretations beyond the stated content.^18 19^ This approach supported our research question and ensured both understanding and transparency within a multidisciplinary research team (medical doctors, general practitioner (GP), critical care nurses, nurse anaesthetists, clinical nutritional experts, surgical ward managers). Reflective thematic analysis proved to be a valuable method for KHar, HVW, ASH, RJT and AS to gain an understanding of patients’ experiences with of using PASC, and to identify the patterns in the data that provided a broader understanding of how PASC influenced patient involvement in safety.^16 19^ During the analysis, the researchers KHar, HVW and ASH re-read all the interviews independently to get a sense of patients’ experiences. Then, following a reflective discussion with HVW and ASH, KHar and HVW revisited the transcripts and composed a written reflection focusing on the individual stories. Next, KHar and HVW coded the data with inductive descriptive codes using words or phrases on experiences or views of PASC. KHar and HVW generated themes from the coding using mind mapping techniques. These themes and maps were discussed in several meetings with all authors. Based on these discussions, maps and the written reflections, KHar, HVW, RJT, AS and ASH finalised the analysis by conceptualising the themes and reporting their content. NVivo V.1.7.1 (1534) was used during the analytical process.^20^ A table presents the analysis with quotations in online supplemental 3. All authors had extensive experience with qualitative analysis.

## Results

31 individual interviews were conducted with both women and men ranging in age from 22 to 88 years. The sociodemographic characteristics are detailed in table 1. Characteristics of both men and women are presented in the table to highlight the diversity in demographics, particularly in terms of surgical specialties.

**Table 1:** Sociodemographics of elective surgical patients (n=31) from the in-depth interviews

	Female	Male	Total
Sex, n (%)	15 (48.4)	16 (51.6)	31 (100)
Age in years, mean (SD)	58.2 (14.9)	65.7 (14.6)	62.1 (15.0)
Days from surgery to interview, mean (SD)	20.5 (10.0)	19.2 (9.9)	19.8 (9.8)
Education, n (%)			
High school	2 (6.5)	4 (12.9)	6 (19.4)
Vocational school	5 (16.1)	4 (12.9)	9 (29.0)
College or university	8 (25.8)	8 (22.6)	14 (45.2)
Work status, n (%)			
Working	9 (29.0)	6 (19.4)	15 (48.4)
Retired/not working	6 (19.4)	10 (32.3)	16 (51.6)
Surgical department, n (%)			
Neurosurgery*	3 (9.7)	1 (3.2)	4 (12.6)
Cardio-thoracic surgery*	1 (3.2)	4 (12.9)	5 (16.1)
Gastrointestinal surgery*	2 (6.3)	3 (9.7)	5 (16.1)
Breast and endocrine surgery	4 (12.9)	1 (3.2)	5 (16.1)
Orthopaedic	4 (12.9)	1 (3.2)	5 (16.1)
General surgery	1 (3.2)	4 (12.9)	5 (16.1)
Ear, nose, throat/Maxillo-facial*	2 (6.5)	2 (6.5)	4 (12.9)
PASC usage, n (%)			
Digital PASC	7 (22.6)	6 (19.4)	13 (41.9)
Paper PASC	5 (16.1)	9 (29.0)	14 (45.2)
Digital/paper PASC	3 (9.7)	1 (3.2)	4 (12.9)
PASC used twice*	–	3 (9.7)	3 (9.7)

* Patients who had surgery at two different surgical departments included in this study and used PASC twice.

PASC, Patients’ Safety Checklist.

**Figure 1:**
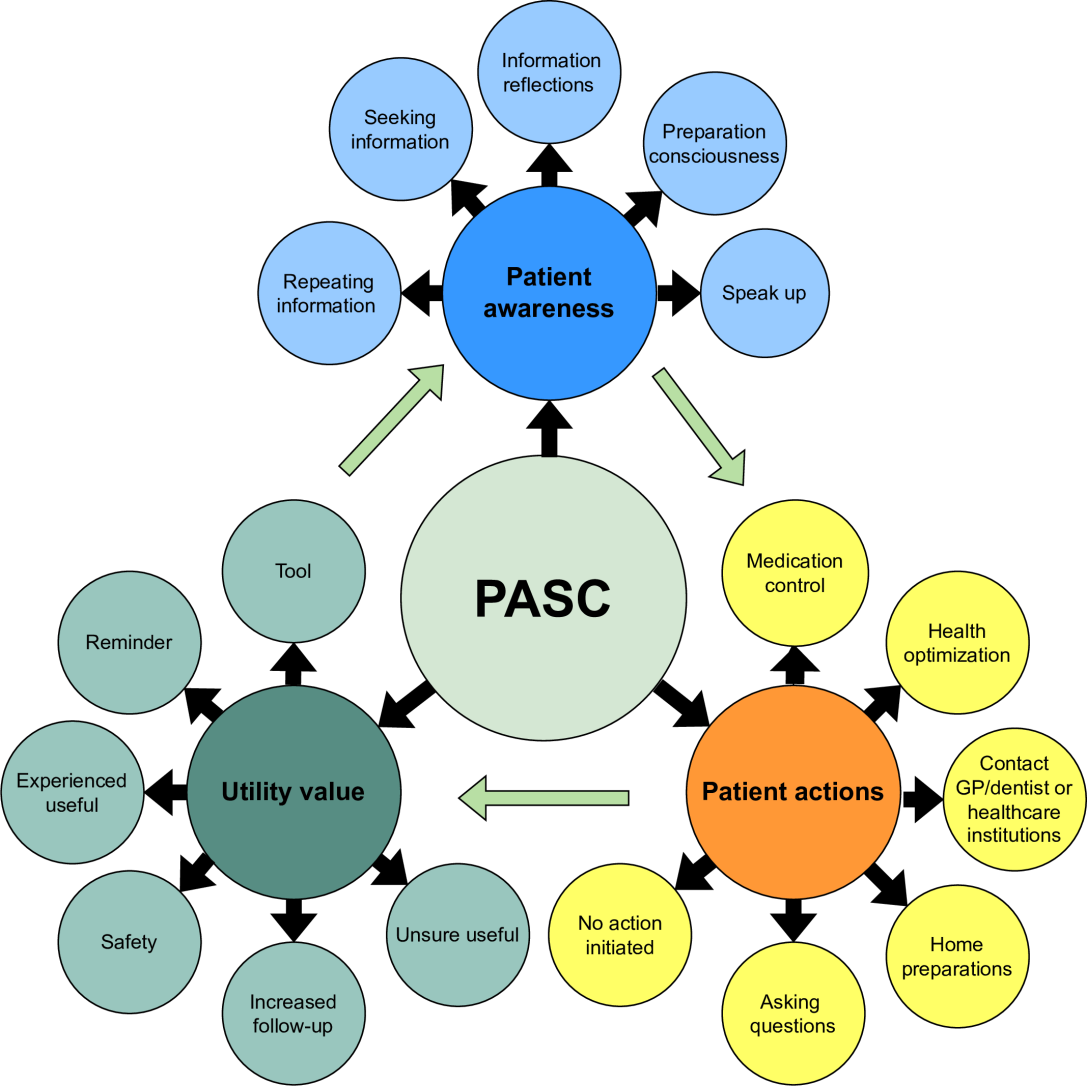
Results of analysis with themes and codes. PASC, Patients’ Safety Checklist.

Three significant themes were generated through the analysis of the data, highlighting patients’ experiences of using PASC throughout their surgical pathway: PASC’s influence on patient awareness, the stimulation of patient actions and its perceived utility value. The first two themes—patient awareness and actions—relate to patients’ preparation before surgery and their recovery at home following discharge. The third theme—utility value—captured the patients’ views on how helpful and meaningful PASC was in supporting their surgical journey. The three themes were based on multiple codes and were conceptually interconnected. The theme of patient awareness encompassed reflective elements that influenced patient actions, while these actions, in turn, reinforced awareness. The dynamic interaction between these two thematic constructs contributed to the third theme, patients’ perceived utility of engaging with the PASC intervention (see figure 1). Selected quotations are included to represent personal insight and diversity within each theme. Participant citations are anonymised; we use ‘P’ for participant and surgical specialty. We observed a general consensus among the participants regarding their experiences with PASC. However, some contrasting views also emerged. These are elaborated under each theme below.

### Patient awareness

This was the first theme generated from the dataset. Patients described that using PASC increased their reflections and consciousness around their own surgical preparations. Patients felt that the PASC items were easy to understand and aligned well with the entire surgical pathway. Their awareness was enhanced regarding each checklist item contributing to reflections over the provided information. This reflective process led them to feel more aware of which preparations they should undertake and increased their understanding of how errors and complications could be prevented. For example, one patient expressed their increased awareness in relation to the content of the PASC items:

When you go through the list (PASC) and answer the questions, you become aware of things. It’s kind of like an ‘aha’ moment, there was a lot I wasn’t aware of, but you don’t really philosophize over it when it’s not your field. P4,Cardio-thoracic surgery

These information reflections, along with the patients’ preparation consciousness, also prompted greater attentiveness to the importance of ‘speaking up’ or ‘voicing your concerns’. Patients mentioned that by actively using PASC, it became easier to recognise when to inform healthcare professionals about crucial information, for example, medical history or medication changes. Other patients mentioned that PASC served as a useful repetition of information. They kept it in the background to cross-check their memory of the information provided. Some took screenshots of the digital PASC list, while others wrote down notes on the paper version.

It was very good to have it in the background. Since it had been a while since I received the verbal information, it gave me the opportunity to repeat the information before the surgery. P1, Breast and Endocrine surgery

In addition, when guiding patients to seek clarifications, PASC also prompted them to find information elsewhere by leading them to trusted sources, for example, hospital webpages, information pamphlets well before surgery or discharge. This again evoked them to be more prepared for presurgical and discharge consultations.

### Patient actions

In the second theme generated from the dataset, patients expressed that using PASC initiated one or more actions to prepare them for surgery or the recovery period.

As described previously, all surgical patients in the trial received the checklist 1–8 weeks before surgery. The patients interviewed stated that they used PASC to prepare for both the surgery and the recovery period. They stated that PASC was used systematically and if information was missing from the hospital or healthcare professionals, the patients would reach out to the appropriate healthcare channels for clarification. Additionally, PASC encouraged them to prepare questions for their pre-surgical and discharge consultations. Two patients expressed it this way:

I got some advice on what I could ask, both before and after (surgery). P 5 General surgery

I received it (PASC) so early that I hadn’t had a conversation at the Hospital, before the operation, I wrote my questions down on a piece of paper and brought them to the meeting (pre-surgical) I had with doctors, nurses, and anesthesia personnel. It gave me a reminder about what I should ask. P1.Gastrointestinal surgery

Further, patients who used medications or had started new medications said that PASC increased their focus on the importance of medication control. This was done by following the medication prompts in PASC, which included learning the names, doses, timings of their medications, understanding their therapeutic areas, ensuring correct medication lists and taking pictures of or bringing the medications/list to the hospital. Patients were activated to contact their GP to update their medication list or to clarify their use of anticoagulants. Others reached out to the hospital to determine when to pause their anticoagulants or other medications, such as antihypertensives or natural medicines. The use of analgesia was also mentioned, patients had asked questions in relation to the PASC items before surgery and others before discharge. The questions were often related to what to take, how often they could take analgesics and what to do if their pain persisted.

Further, patients mentioned that they were aware of the importance of optimising own their health and maintaining a healthy lifestyle prior to surgery. However, they thought this PASC item was valuable and stated that it enhanced their focus on a healthy diet and exercise presurgery. Others mentioned that the item addressing dental health was very useful and one patient contacted the dentist which revealed an infection:

I injured a tooth when I was younger, and then he (dentist) found out that I had an infection. He said it wasn’t dangerous, but that the tooth needed to be fixed, which I didn’t know about. I’m very glad that I followed this recommendation (item in PASC). P4, General surgery

Other patients also mentioned that most of the PASC items were already covered by healthcare professionals, so they did not need to take any additional actions. They viewed PASC more as a safety check before and after surgery. Others, with extensive experience from previous surgeries, felt that the content of PASC was familiar and the items did not provide any new information to them.

They covered it themselves (healthcare professionals) the information was in line with the checklist, I did not have to ask (additional questions). P1,Ear, Nose, Throat /Neurosurgery

### Utility value

This was the final theme generated from the dataset. This theme related to how patients perceived PASC as valuable. Patients experienced PASC as important and useful. They stated that they would use PASC if they were to undergo another surgery. The patients confirmed PASC to have utility value often referred to as a ‘tool’. They described it to systematise, provide additional health information and allow them to review information throughout their surgical pathway. One patient reflected over this in relation to elderly patients:

I also think of elderly people who are insecure, so I believe this is a very useful tool for them. P2, Orthopedic surgery

In addition, patients mentioned that having PASC as a reminder in the background gave them a sense of safety, as it allowed them to ensure that all information and preparations were covered, understood and remembered. One patient expressed this:

I think it provides a sense of security. I actually gained a better understanding, right! For me, I have to say. P4, Cardio-thoracic surgery

One positive aspect of such a checklist is that it increases awareness that you are going to undergo a surgery, which can lead to complications if you do not follow the guidelines. Just by receiving information through a questionnaire (PASC), you become aware that it is important to follow the guidelines you have been given. In this way, it is both informative and preventive. P3, Orthopedic surgery

Other patients used PASC in conjunction with a healthcare professional. These patients reported feeling extra cared for or followed up, because PASC promoted communication and clarification. However, patients also used PASC independently, and they stated that PASC increased the sense of being followed up throughout their surgery.

Two patients stated they were unsure if PASC was useful; they thought it did not give anything new. This was information they had either already received or had existing knowledge about from previous surgical experiences. However, these patients also expressed the view that they would use PASC again if they had to have another surgery.

## Discussion

This study shows that patients generally had positive experiences with PASC. They found the checklist valuable for their surgical care and safety. The checklist was described as a systematic tool that increased their awareness regarding preparation and important information, which led to patient actions. These actions were carried out to ensure that they had control over their own surgical care and how they could do to ensure their own safety. Based on this and the literature on patient-driven checklists, such interventions should be given more attention within today’s healthcare organisations.^1021^,^23^ PASC is novel in its development, design and purpose. There are few existing patient-driven checklists like PASC that have been rigorously developed and researched over time, and there is a strong indication that its implementation can enhance patients’ involvement in quality of care and safety.^9^

Further, our findings suggest that, from a patient perspective, PASC is a valuable contribution towards increasing patients’ surgical safety involvement, which is in line with WHO’s vision to eliminate all preventable harm and the newly published patient safety rights charter.^5 6^ PASC is designed as a multifaceted tool for patients to use, and our findings indicate that it enhances their ability to navigate health information and actively engage in safety practices. This is achieved by raising awareness of critical issues, thereby encouraging actions that may help reduce the risk of errors and complications. There are currently few existing patient-completed checklists.^7^,^10^ To our knowledge, PASC is the only checklist designed for multiple surgical specialties and that follows the patients throughout the whole surgical pathway.^11^ Surgical safety encompasses a timeline that begins weeks or even months prior to the operation and continues well beyond the patient’s discharge from the hospital.^23^ During this period, patients often experience numerous care transitions, and a literature review from 2013 concluded that there were more preventable errors occurring during this period before and after surgery than during the surgical procedure itself.^24^ The review concluded that further safety improvement initiatives should target the whole surgical pathway.^24^ Systematic progress in this area has been slow regarding patient involvement in their own safety and we are still facing the same challenges today.^3 25^ Medical errors followed by healthcare-associated infections and patient care events remain at the top of the list of preventable mistakes in the surgical setting.^25^ Hence, the call for patient-driven checklist^26^ and the surgical patients’ experiences with using PASC may be a valuable and important contribution to reduce such events.

As mentioned earlier, the patients found PASC to be of high utility value for surgical preparations. Their view of utility value initiated them to use and follow the instructions within PASC throughout the surgical pathway. PASC covers an extensive area of risks.^11^ The surgical patients experienced that by using PASC, they could contribute to eliminating risks. These risk areas were: medication errors, optimisation of own health, including nutritional status, home and surgical preparations, awareness about safety measures just before surgery, which is encompassing the use of safe surgery checklist, and lastly the importance of discharge information. Each item on PASC ensures that the patients know when and who to contact (eg, GP, dentist or hospital) when information, instructions, etc are missing or unclear.^11^ Our findings suggest that PASC strengthens patients’ understanding of risk factors and empowers them to make informed decisions, which is perceived to contribute to improved patient safety.^26^ However, PASC’s full effect on preventable errors related to surgical procedures is not fully explored yet, a clinical trial assessing its effect on complications and hospitalisation length is underway.^14^

PASC was initially designed for patient use. However, results from this study and our previous PASC study^12^ indicate that PASC might be useful for healthcare professionals as well. Especially when it comes to ensuring that patients receive and understand information.^11^ Our study found that when PASC was used in conjunction with healthcare professionals, it increased the patient’s sense of care and safety, which emphasises the importance of patient—healthcare organisation and professionals’ collaboration. The need for such interventions is also emphasised in the current literature.^3 22 25 26^ In addition, if PASC is implemented as a collaboration tool used actively from both patients and healthcare professionals, it might create a patient involvement safety culture that is more open for engaging patients in their own safety. On the other hand, our results also indicate that PASC encourages patient involvement by giving clear and understandable instructions; in this manner, they might also be more aware of their responsibilities in relation to preparing for surgery and ensuring their safety. Several recent studies on patient involvement in safety are calling for such interventions to be developed and implemented at organisational and policy level.^23 27^ This further aligns with WHO’s recommendations on patients’ rights.^5^ Patient-driven checklists are identified in the literature as an important step in this direction^21 22^ and also as a potential facilitator in activating patients’ contributions within the wider care team.^21 22^ Furthermore, PASC is identified in a recent scoping review of 32 studies to be one out of two patient involvement interventions that could be implemented at both organisational and policy levels due to its rigorous development and validation process with patients’ participation.^24^ Considering findings from this and our previous studies, we suggest that PASC is a step towards empowering patient involvement in surgical safety. We recommend other countries adopt PASC to their surgical settings.

### Strengths and limitations

Study limitations include the potential for recall bias in the data collection, since some patients were interviewed up to 5 weeks after surgery. However, some of these patients had used PASC twice in two different surgical departments and in general, most of the participants stated that they had a positive experience with using PASC. Another limitation could be that KHar and ASH were the main developers of PASC and conducted interviews, which could lead to favour bias in relation to the data collection. The addition of other researchers was an attempt to minimise this risk. HVW and RJT participated in the data collection, and HVW, RJT and AS participated in the analysis process. It also must be mentioned that PASC development and testing have been carried out in a Norwegian setting and it remains to be investigated how patients from other healthcare systems and cultures experience its use.

Strengths of this study include the variety of strategically sampled surgical patients participating across seven surgical specialties and three hospitals. This ensured a wide geographical and equal gender representation within the sample, strengthening the findings. Second, patients were individually interviewed by three researchers (ASH, KHar and HVW). The individual interviews were discussed within this team, which gave the researchers a sense of validity regarding the consensus within the data.^18^ Lastly, the findings of this study are in line with previous qualitative findings from a PASC feasibility study, where three focus group interviews were carried out.^12^

### Rigour

Throughout our analysis, we ensured rigour by following Lincoln and Guba’s quality criteria—credibility, transferability, dependability, confirmability and authenticity.^28^ To achieve credibility, participants were strategically selected to ensure information power, according to Malterud *et al*,^29^ thereby representing the experiences of patients from all surgical specialties included in the SWCRCT. In addition, triangulation was supported by the wide variation in participants’ backgrounds and the involvement of a multidisciplinary research team, which was active throughout the entire research process—from planning the study to reporting the results.^18^ The use of a multidisciplinary team and informants from seven surgical specialties ensured rich data, and our findings reflected both positive and negative patient perspectives, supporting the study’s transferability.^28^ By adhering to Braun and Clarke’s methodological guidelines, we ensured transparency in coding and theme development, thereby enhancing the study’s dependability, confirmability and authenticity.^18^

## Conclusions

PASC is a patient-driven checklist that patients perceive as an important tool to enhance their involvement in surgical care and safety. PASC provides patients with an opportunity to systematically reflect over the importance of surgical preparation and information throughout the surgical pathway. Implementation of a multifaceted tool like PASC might ensure systematisation of information across surgical settings, correct information and patient instructions, enhanced patient–healthcare professional communication and patient involvement. Further research is needed on implementing and adapting PASC to other clinical settings.

## Supplementary material

10.1136/bmjopen-2025-105554 online supplemental file 1

10.1136/bmjopen-2025-105554 online supplemental file 2

10.1136/bmjopen-2025-105554 online supplemental file 3

## Data Availability

Data are available on reasonable request.
