# Supplementary material for: Surgical patients’ experiences with the Patients’ Safety Checklist (PASC): a qualitative interview study
Source: BMJ Open. 2025 Oct 5;15(10):e105554. doi: 10.1136/bmjopen-2025-105554 (PMC12506218; doi:10.1136/bmjopen-2025-105554)
Supplement: online supplemental file 2 [file bmjopen-15-10-s002.docx]

**Patient Interview Guide (Translated from Norwegian to English)**

**Opening Questions:**

**Introduction question 1**: Education. Employment. Time of surgery.

**Introduction Questions 2**: Can you briefly share your experiences with using the patient checklist?

**Supporting questions:**

-How was it to use?

-How did you use it? (paper vs electronic - mobile, tablet)

-When did you use it – filled out all at once/over time? (time spent, understandable SMS, helsenorge (Norwegian digital health platform), understandable language, readable writing, print list/screenshot?)

-Time of receipt of the checklist?

-Structure of the checklist – order of items natural, difficult to answer, other alternatives?

-Did healthcare personnel at the hospital ask you about the checklist - if you had used it, had questions, and possibly the content?

**Transition Questions 3**: What was there any positive aspect for you with using the checklist?

-What could have been done differently? Other points? Reason?

**Question 4**: Did the checklist clarify misunderstandings or prevent complications for you, if so, which ones?

-Did you experience any misunderstandings, errors, and delays/cancellations (undesirable events)? Before and after surgery. Could it have been avoided with checklist items?

**Question 5:** How did the checklist affect the information you needed before the surgery?

-Did you request information because of items on the checklist?

-From whom or where did you get information (relation to the checklist)?

-How was it to bring up things (communication) with the nurse, doctor, and surgeon?

-Did you get clarifying answers to your questions?

**Question 6.a:** How did the checklist affect your preparation for surgery?

-Did you make any lifestyle changes (smoking, alcohol, diet, exercise)?

-Did you learn the names and appearance of the medications you use?

-Did you contact your GP or dentist?

-Did you clarify if someone could be home/have things ready when you got home, or if you needed rehabilitation/physiotherapy?

**Question 6.b:** The checklist has questions about nutrition – how was it to fill out questions about weight and food intake?

-Was it relevant to contact a nutritionist?

**Question 7**: How did the checklist affect the information you needed after the surgery?

(Did you request information because of checklist items)

-From whom or where did you get information? (nurse, surgeon, GP, others?)

-Did you get clarifying answers to your questions?

-Did you need more, if so, what information after returning home?

**Question 8:** How did the checklist affect your preparation for discharge from the hospital?

-How well prepared were you regarding the topics on the checklist (complications, activity and restrictions, medications, painkillers, bowel function, further plan and follow-up)?

-What measures did you take because of the checklist?

**Question 9:** How did you experience using the checklist? Did you feel safe or unsafe using the checklist?

-Did you find it useful to use? Why/why not?

**Question 10:** Would you choose to use a patient checklist for a new surgery? If so, why?
