# Supplementary material for: Surgical patients’ experiences with the Patients’ Safety Checklist (PASC): a qualitative interview study
Source: BMJ Open. 2025 Oct 5;15(10):e105554. doi: 10.1136/bmjopen-2025-105554 (PMC12506218; doi:10.1136/bmjopen-2025-105554)
Supplement: online supplemental file 3 [file bmjopen-15-10-s003.docx]

**Supplement 2.** Coding table

| Theme | codes | Examples |
| --- | --- | --- |
| Patients’ awareness | Information reflections  Preparation consciousness  Speak up  Seeking information  Repeating information | *“I had to stop filling the checklist out because there were things I had to follow up. It was not the checklists fault it was related to my literacy level”* **P4, General surgery**  *“I think the positive thing is that it is a way to prepare people for what is going to happen. I believe that people have a great need for information when they are going to be admitted (to hospital). There are probably a lot of concerns around that. I thought it was a good initiative to get such a list (PASC) in advance. You can then take some time to reflect and respond. So I would say, for my part, I think it is a good initiative."* **P2, Neurosurgery**  *"You need to gather the courage to clearly tell the doctor that you don't understand this, that it doesn't seem right, and you need to stand your ground."* **P4, General surgery**  ***"I felt like PASC gave me some clarity on what I should thinking about —it simply made me more aware."* P1, Ear, nose, throat surgery** |
| Patients’ actions | Medication control  Health optimization  Contact GP/Dentist or other healthcare institutions  Home preparations  Asking questions  No action initiated | *“I got a dental appointment, so he could check my teeth, which I’m very glad for. I had great benefits of using this checklist”* **P4, General surgery**  *“I prepared my medication list and brought it with me to the hospital”* **P1, Neurosurgery**  *“I could take notes, then I took a screenshot of the remembering list that came up at the end of the checklist, I actually used it when I talked to the surgeon before I was discharged”* ***P2,* Cardio-thoracic surgery**  *“I clarified everything regarding home transport, because I have long way to travel (several of hours) and also what I needed to have at home”* ***P*3, Orthopedic surgery**  *“I live alone and had to ensure that someone was with med the first day after discharge”* **P5,** **Breast endocrine surgery**  “*There were a few questions I written down and should have asked, but the discharge process was rushed”* **P1, Breast endocrine**  *“I did not need to clarify any questions because I have had an operation before. I knew the routines and what I had to go through”* ***P5,* Cardio-thoracic surgery** |
| Theme | Codes | Examples |
| Utility value | A tool  Reminder  Experienced useful  Safety  Increased follow-up  Unsure useful | *The checklist was in a way a repetition of information and then there was some new information. I thought it was very good, the checklist caught what was necessary to repeat, information that I had received earlier and forgotten, so this was very good”* **P4, *General surgery***  *“It was useful, but it could have had even more use of the one after surgery if had the opportunity for a better conversation with the healthcare personal”* **P2, Ear, nose, throat surgery**  *"No, I think they give people a sense of security, I believe. You become more aware of what you need to think about and remember. There's something about starting the thought process before an operation. I think that's just positive regardless. The more you know and have thought about in advance, the better it is afterwards"* **P1, Neurosurgery**  *“I have been through some of this before, so it was not that useful for me. I did not need it (PASC)”* **P4, Orthopedic surgery** |
