# Supplementary material for: Surgical patients’ experiences with the Patients’ Safety Checklist (PASC): a qualitative interview study
Source: BMJ Open. 2025 Oct 5;15(10):e105554. doi: 10.1136/bmjopen-2025-105554 (PMC12506218; doi:10.1136/bmjopen-2025-105554)
Supplement: Supplementary file 4 [file bmjopen-2025-105554.reviewer_comments.pdf]

PEER REVIEW HISTORY

BMJ Open publishes all reviews undertaken for accepted manuscripts. Reviewers are asked to complete a checklist review form and are provided with free text boxes to elaborate on their assessment. These free text comments are reproduced below.

ARTICLE DETAILS

Title (Provisional)

Surgical patients' experiences with the Patients' Safety Checklist (PASC) – a qualitative interview study.

Authors

Harris, Kristin; Valen Wæhle, Hilde; Storesund, Anette; Harthug, Stig; Tangvik, Randi Julie; Monsen Lukcova, Denisa; Håvik, Wenche; Humberset, Åse; Stavang, Evelyn; Hagerup, Kjetil; Teigland Tepstad, Åshild; Sandsbakk Austarheim, Ann Kristin; Healey, Andy; Sevdalis, Nick; Haugen, Arvid

---

VERSION 1 - REVIEW

**Reviewer**	**1**
**Name**	**Schandl, Anna**
**Affiliation**	**Karolinska Institute, Clinical Sciences and Education Södersjukhuset**
**Date**	**30-Jun-2025**
**COI**	**None**

Thank you for inviting me to review this interesting interview study about the experiences of using a patient safety checklist. Involving patients in safety procedures is rather unique and deserves to be highlighted. The manuscript is well-written, and the results are interesting and should be read by clinicians involved in surgical care. However, I do have some questions and suggestions about the study:

Methods:

In my opinion, this interview study is purely of a qualitative descriptive study design and not prospective. Please, remove the term "prospective" (line 106).

Could you please provide a brief summary of the PASC trial? While it is mentioned in passing, it would strengthen the context of the interview study to include the trial's objective and primary outcome measures.

The recruitment process needs to be elucidated. It currently reads ….. from which patients were recruited into this study by the wards themselves….? What does this mean? (lines 122-123)

Why is Table 1 categorised into females and males? Are the potential sex differences of importance?

In qualitative research, it is more customary to state that" no participants withdrew from the study" rather than "no participants dropped out during the study". Therefore, I recommend revising the sentence to align with standard academic phrasing in qualitative reporting.

Results:

In qualitative studies, it is generally avoided to use quantifying terms such as "most" or "several patients", as the aim is not to measure frequency but to explore the breadth and depth of individual experiences. The focus lies in capturing the variation and richness of perspectives rather than determining how commonly a particular view is held. Therefore, I recommend revising the results section to reflect this approach, emphasising the diversity of experiences rather than their prevalence.

Overall, the results are interesting. I do like the summary at the beginning of the discussion. However, this section does not clearly reflect the results. Is it possible to clarify the results in line with your conclusion? All main themes start with describing the codes, except the third. Why are they not included there? In theme 2, home preparedness and discharge preparation are not described in the text – and the latter is absent in the figure. Please revise where needed.

Discussion:

Please, include a discussion of the study's limitations, specifically addressing the criteria of credibility, dependability, confirmability, and transferability, which are essential for evaluating the trustworthiness of qualitative research.

A minor comment, some words are missing in line 349 (currently reads, an attempt to minimise this risk HWV in the data collection ……)?

**Reviewer**	**2**
**Name**	**Karampatakis, Georgios Dimitrios**
**Affiliation**	**Queen Mary University of London, Wolfson Institute of Population Health**
**Date**	**14-Jul-2025**
**COI**	**None**

The topic could be of interest bearing in mind the complexity of patients in surgical wards. Unfortunately, the manuscript is not written to a standard that can be published, there are significant flaws throughout especially when it comes to data analysis and reporting of findings. Data analysis needs to be repeated; it seems that very little analysis has been performed and only a few scattered ideas/codes are currently being presented. The whole manuscript should be re-written. The use of English language is problematic throughout, to the point that readers can only assume/guess the intended meaning, there are countless errors with the use of prepositions and many other grammatical mistakes and mistakes with the use of vocabulary. Apologies for any disappointment caused, hope my comments help in the process of improving the manuscript.

-Title: Please check grammar, i.e., it should read patients'… not patient's. Also state the type of the study, e.g., a qualitative interview study.

Abstract:

-Lines 27-33: Reading the Background I am not sure I grasp the complete problem. E.g., Engaging patients in what way and how? What sort of preventable events authors are referring to? Is this study part of the Stepped Wedge Cluster Randomized Controlled Trial? State more explicitly, rather than 'this trial'.

-Lines 34-38: What does 'elective surgical patients' mean? How were participants recruited? Where did the interviews take place? Were the interviews one-to-one?

-Lines 39-44: As a reader, and despite being in healthcare, I understand nothing from the Results in the Abstract. I appreciate that it is just an abstract, but themes need to be explained more clearly. E.g., awareness of what? Actions about what? What does 'utility value' mean?

-Lines 45-50: 'preparedness for surgery and the time after', what does preparedness for the time after mean? What does 'other clinical settings' mean? Isn't this checklist just for surgery-related patients?

-Lines 53-64: Please stick to the headings in here. What is already known on the topic should just state existing knowledge. What is the existing knowledge? What does 'proactive steps' mean?

Background

-Lines 71-80: You will need to provide examples of preventable adverse events. Having read this paragraph, I am not sure where do these checklists come into play? At what stage and how? Who monitors them? Who introduces them? What is the ultimate goal and what patients are supposed to do?

-Lines 81-104: What does 'safety researchers' mean? What does 'elective surgery' mean? There is no reference whatsoever to existing knowledge and literature. What do we already know about checklists like such and where does this study come into play? Just stating there

is a knowledge gap says nothing and makes me question, as a reader, the gaps in knowledge this study is attempting to fill in. What does ‘post-surgical patients’ experiences’ mean? What does the ‘surgical pathway’ mean?

Methods:

-Interviews were one-off, weren’t they? How could this study be a prospective one?

-You will need to provide some more detail about the Stepped Wedge Cluster Randomized Controlled Trial. What were participants supposed to do?

-Remove any reference to COREQ in the main text, this is just for the review process.

-‘Mentally and cognitively healthy’, how this was determined and by whom?

-What does fluent in ‘intervention arm’ mean? You can only be fluent in a language.

-Lines 127-129 and Table 1 is Results rather than Methods. You wouldn’t know in advance how many would have participated. Also, did you carry on with recruitment until data saturation?

-Was there any sort of recruitment material used? How were the interviews scheduled? Were any reminders sent?

-Lines 132-150: Were the interviews in person or remote? Who carried out the interviews and what experience of qualitative research did they have? Were interesting topics arising during data collection explored in subsequent interviews? It is not a great practice having a ‘rigid’ topic guide in qualitative research. The fact that there was no prior relationship between researchers and participants makes me questioning the honesty of participants’ views, how was rapport established between a bedridden and in discomfort (presumably) patient in a surgical ward and a researcher they never knew?

-Lines 146-148: I don’t think there is a lot of appreciation as to what ‘data saturation’ mean. Data saturation usually has recruitment implications. You will need to state the precise model of saturation used and what this meant for recruitment.

-Lines 148-250: I totally do not understand these sentences. Do you mean asking researchers to exclude their data? Otherwise they wouldn’t have been able to drop out, this was only a one-off interview (it is a black and white situation).

-Lines 153-159: I am sure I fully understand how exactly data analysis was done. How did you move from codes to categories, then to themes? Was there any framework used (e.g., Braun and Clarke) to guide the analysis process? What you present in Supplement 3 is not a coding tree. Quotations cannot be presented twice. Were there any disagreements amongst researchers when it came to data analysis? How these were resolved, if so?

Results:

-Line 164: if themes are closely interconnected this means that analysis was not properly done, themes need to be mutually exclusive!

-Lines 168-170: Remove these sentences, they say nothing to the reader.

-Lines 173-176: Apologies, this is not how qualitative research is being reported. Results present the themes, not the codes.

-Apologies I am completely unable to follow the Results. It seems that very limited analysis has been performed and I am unable to comment any further. It seems that what is being currently presented is just a few scattered codes/concepts without any higher level of synthesis. Quotations are not being presented properly (e.g., after the quotation it is imperative to state Participant 1, 2, 3 etc., you can then state the ward if you want and all this should be presented in brackets). Quotations just repeat the narrative without providing any new data. The whole analysis should be repeated so that findings are presented succinctly and as per the standards of qualitative research.

Discussion:

-Once more apologies. Having not understood the Results, it is impossible to comment on the Discussion. At the moment arguments are all over the place and difficult to follow. Discussion sections should have the following structure: brief summary of findings within the first paragraph, comparison with existing literature in the next few paragraphs (each paragraph should be succinct and should flow well from the previous one), then a section about implications of the study before moving To Strengths and Limitations.

-Strengths and Limitations: arguments are not clear and I can only assume what the implied meaning is. E.g., 'another limitation could be that KH and ASH were the main developer of PASC and also interviewers', why is this a limitation? What sort of bias you are referring to? Please re-write this section in a clearer and more succinct way.

VERSION 1 - AUTHOR RESPONSE

Reviewer 1 comments:

Methods:

1.- In my opinion, this interview study is purely of a qualitative descriptive study design and not prospective. Please, remove the term "prospective" (line 106).

Thank you for your remark, we have removed the term "prospective" and performed adjustments to the text on page 3, line117-118:

"This was an exploratory qualitative study, based on in-depth individual patient interviews."

2. *Could you please provide a brief summary of the PASC trial? While it is mentioned in passing, it would strengthen the context of the interview study to include the trial's objective and primary outcome measures.*

Thank you for highlighting this we have added a brief summery under the method section page 3, line 118-125:

“The study is a part of a Stepped Wedge Cluster Randomized Controlled Trial (SWCRCT) (14), designed to investigate the impact of PASC on elective surgical complications, LOS in hospital, and mortality, as well as patients’ experiences with using PASC. Data in this trial were collected from November 2021 to February 2024. The elective surgical patients in the control group received care as usual, while the patients in the intervention group received PASC both electronically and by mail, one to eight weeks before surgery. (142). In this part of the study, participants were asked to share their experiences using PASC throughout their surgical pathway.”

3. *The recruitment process needs to be elucidated. It currently reads ….. from which patients were recruited into this study by the wards themselves….? What does this mean? (lines 122-123)*
   Thank you highlighting this, we have amended the text to following page 4, line 139-141:

   “Seven surgical specialties across eight surgical wards participated in the SWCRCT, from which patients were recruited by the healthcare professionals at each ward”

   We have also further elaborated the text on the recruitment process to ensure clarity under section Settings and Participants, page 4, line 147-152

   “Patients enrolled in the trial were strategically sampled to participate in in-depth interviews post-discharge. They were invited either before discharge from the hospitals or contacted after discharge via phone by ASH, KH, HWV, and RJT. If they accepted the invitation, an interview appointment was scheduled. No reminders were sent out. ASH, KH and HWV carried out the phone interviews, from a hospital office phone.”

4. *Why is Table 1 categorised into females and males? Are the potential sex differences of importance?*

   Thank you for this comment. Based on Braun and Clark Reflexive Thematic Analysis Reporting Guidelines (RTARG) and their book on Thematical analysis we have decided to keep the table as it is to give a clear representation of the variation of the socio-demographics in our participants. However, the table is moved to the result section based on reviewer 2’s comments on page 6 Line 205. We have also altered the text before the table to meet the RTARG guideline on page 5, line 201-204:

   “Thirty-one individual interviews were conducted with both women and men ranging in age from 22 to 88 years. The socio-demographic characteristics are detailed in Table 1.

Characteristics of both men and women are presented in the table to highlight the diversity in demographics, particularly in terms of surgical specialties."

5. *In qualitative research, it is more customary to state that" no participants withdrew from the study" rather than "no participants dropped out during the study". Therefore, I recommend revising the sentence to align with standard academic phrasing in qualitative reporting.*
   Thank you for your remark we have now changed this sentence page 4, line 152:

   "No participants withdrew from the study."

   Results:
6. *In qualitative studies, it is generally avoided to use quantifying terms such as "most" or "several patients", as the aim is not to measure frequency but to explore the breadth and depth of individual experiences. The focus lies in capturing the variation and richness of perspectives rather than determining how commonly a particular view is held. Therefore, I recommend revising the results section to reflect this approach, emphasising the diversity of experiences rather than their prevalence.*
   Thank you for your remark we have now removed most of these terms in the result section, except one page 9 line 303. The reason for this is that Braun V, and Clark V. Thematic Analyses: A practical guide, SAGE publications Inc. 2022 page 142, state the following: "some quantifying can be used to discuss its prevalence across the data corpus"

7. *All main themes start with describing the codes, except the third. Why are they not included there? In theme 2, home preparedness and discharge preparation are not described in the text – and the latter is absent in the figure. Please revise where needed.*

   Thank you so much for discovering this detail, we have actually removed all the descriptive codes from the result section, due to the second reviewers' comments and after reading up on the latest publication from: Braun V, Clarke V. Supporting best practice in reflexive thematic analysis reporting in Palliative Medicine: A review of published research and introduction to the Reflexive Thematic Analysis Reporting Guidelines (RTARG). Palliative Medicine. 2024;38(6):608-616. doi:10.1177/02692163241234800

6. *Overall, the results are interesting. I do like the summary at the beginning of the discussion. However, this section does not clearly reflect the results. Is it possible to clarify the results in line with your conclusion?*

Thank you for your, remark we have now re-written parts of the discussion to ensure that they are in-line with our findings and conclusion please see the discussion section on page 10, line 356-359, page 10 line 373-380, page 11, line 385-387 and page 396-398.

Discussion:

9. *Please, include a discussion of the study's limitations, specifically addressing the criteria of credibility, dependability, confirmability, and transferability, which are essential for evaluating the trustworthiness of qualitative research.*

   Thank you for comment, we have now added a new section Rigor according to Braun and Clarke's Reflexive Thematic Analysis Reporting Guidelines on page 12, line 436-448:

   "Throughout our analysis, we ensured rigor by following Lincoln and Guba's quality criteria—credibility, transferability, dependability, confirmability, and authenticity (28). To achieve credibility, participants were strategically selected to ensure information power, according to Malterud et al. (29), thereby representing the experiences of patients from all surgical specialties included in the SWCRCT. In addition, triangulation was supported by the wide variation in participants' backgrounds and the involvement of a multidisciplinary research team, which was active throughout the entire research process—from planning the study to reporting the results (18). The use of a multidisciplinary team and informants from seven surgical specialties ensured rich data, and our findings reflected both positive and negative patient perspectives, supporting the study's transferability (28). By adhering to Braun and Clarke's methodological guidelines, we ensured transparency in coding and theme development, thereby enhancing the study's dependability, confirmability, and authenticity (18)."

10. *A minor comment, some words are missing in line 349 (currently reads, an attempt to minimise this risk HWV in the data collection ......)?*
    Thank you for seeing this. We have now amended the text in the following page 11, line 424.
    "HWV and RJT participated in the data collection"

Reviewer 2 comments:

1. *Title: Please check grammar, i.e., it should read patients'... not patient's. Also state the type of the study, e.g., a qualitative interview study.*
   Thank you for your remark, *patient's* has been changes to *patients'* and the qualitative interview study has been added to the title, page 1 line 1-2:

   "Surgical patients' experiences with the Patients' Safety Checklist (PASC) –
   a qualitative interview study."

Abstract:

2. *Lines 27-33: Reading the Background I am not sure I grasp the complete problem. E.g., Engaging patients in what way and how? What sort of preventable events authors are referring to? Is this study part of the Stepped Wedge Cluster Randomized Controlled Trial? State more explicitly, rather than 'this trial'.*

   Thank you for your comment. We have elaborated that the study is part of the SWCRCT in the abstract; background section page 2 line 43-47:

   "A Patients' Safety Checklist (PASC) has been developed and validated for use by surgical patients. It's effect on patient safety and patient outcomes is currently being investigated in a Stepped Wedge Cluster Randomized Controlled Trial (NCT03105713). In connection with this trial, we have examined elective patients experiences with using the PASC."

   and described more in detail on the preventable events under the background section in the manuscript on page 2, line 88-83:

   "Despite the implementation of surgical safety checklists and other safety measures in surgical specialties (2,3), preventable adverse events (e.g., medication errors and healthcare associated infections) continue to occur and further research is warranted (4)."

   However, the authors do not full agree with your comments here, and argue that: 'Engaging patients in surgical safety' is discussed widely in published papers within the field of surgery, and also used by the WHO. Hence, we choose to use this term without any further justifications.

3. *Lines 34-38: What does 'elective surgical patients' mean? How were participants recruited? Where did the interviews take place? Were the interviews one-to-one?*

   Thank you for your remark. "Elective surgical patients'" is a common terminology used in the surgical field and is defined as a MESH term as elective surgical procedures. As author within this field, we do not see the need to further clarify this term. However, your valuable comments recruitment have been taken into consideration and the following amendments have been carried out in the abstract on page 2, line 48-49:

   "An explorative qualitative study based on individual in-dept phone interviews with 31 elective surgical patients."

And under the settings and participant section on page 4, line 151-152.

"ASH, KH and HWV carried out the phone interviews, from a hospital office phone."

4. *Lines 39-44: As a reader, and despite being in healthcare, I understand nothing from the Results in the Abstract. I appreciate that it is just an abstract, but themes need to be explained more clearly. E.g., awareness of what? Actions about what? What does 'utility value' mean?*

   Thank you for your remark, we have changed the word reflection to awareness in the sentence under the themes to ensure consistency on page 2, line 54 and 56.

   In addition the theme content has been described in more detail to ensure clarity on page 2 lines 53-59:
   "Three themes were identified in the data: Patient awareness, Patient actions and Utility value. Patient perceived PASC to increase awareness around surgical information, preparations, what to speak up about, and which information to seek and repeat. This awareness led to a series of actions, such as ensuring medication control, optimalization of own health, contacting healthcare professionals, asking questions and, for some no actions were needed. Patients perceived PASC to have high utility value for their surgical preparation."

5. *-Lines 45-50: 'preparedness for surgery and the time after', what does preparedness for the time after mean? What does 'other clinical settings' mean?*
   Thank you for comment, we have changed the 'time after' to 'recovery' in the abstract section on page 2, line 62-63:

   "PASC is one of few existing interventions that specifically focus on assisting patients in preparing for surgery and managing their recovery"

   'Clinical settings' is a common term used in the medical field: Gellman. M.D, Turner R.J. (2013) Encyclopaedia of Behavioural Medicine. Springer, New York, NY. https://doi.org/10.1007/978-1-4419-1005-9_94. Authors have therefore not amended this term.

   Isn't this checklist just for surgery-related patients?

   Thank you for this comment, yes, it is but our ongoing research suggests that patient-driven checklists should also be adopted in other medical settings, such as the secondary care.

6. *-Lines 53-64: Please stick to the headings in here. What is already known on the topic should just state existing knowledge. What is the existing knowledge? What does 'proactive steps' mean?*
   Thank you for your remark this is section is now removed.

Background

7. *Lines 71-80: You will need to provide examples of preventable adverse events. Having read this paragraph, I am not sure where do these checklists come into play? At what stage and how? Who monitors them? Who introduces them? What is the ultimate goal and what patients are supposed to do?*
   Thank you for your remark, authors do not fully agree with this comment. Within the safety field preventable adverse events are commonly known and has hardly changed over the last decade, please see reference: *Bates DW, Levine DM, Salmasian H, et al. The Safety of Inpatient Health Care. N Engl J Med. 2023;388(2):142-153. doi:10.1056/NEJMsa2206117*

   However, we acknowledge that some examples of preventable adverse events may help the reader to understand what the challenges are, and have altered the text under background on page 3 line, 80-83

   "Despite the implementation of surgical safety checklists and other safety measures in surgical specialties (2,3), preventable adverse events (e.g. medication errors and healthcare associated infections) continue to occur and further research is warranted (4)"

   Further, here the authors refer to checklists in general and not the PASC specifically.

   PASC is described in more detail on page 3, line 91-111 (original manuscript)

   The use of checklists and other safety measures have been widely discussed both in the literature and by WHO. We have added two more references to elaborate this under the background on page 2, line 82. The following reference were added the other was moved up from the discussion section: Haynes AB, Weiser TG, Berry WR, et al. A surgical safety checklist to reduce morbidity and mortality in a global population. N Engl J Med. 2009;360(5):491-499. doi:10.1056/NEJMsa0810119

8. *Lines 81-104: What does 'safety researchers' mean? What does 'elective surgery' mean? There is no reference whatsoever to existing knowledge and literature. What do we already know about checklists like such and where does this study come into play? Just stating there is a knowledge gap says nothing and makes me question, as a reader, the*

gaps in knowledge this study is attempting to fill in. What does 'post-surgical patients' experiences' mean? What does the 'surgical pathway' mean?
Thank you for your remark. Safety researcher is a common terminology in our field of research, here is a description: A safety researcher investigates factors that can cause harm or injury and develops strategies to prevent or mitigate these risks. We have therefore not changed this or explained it further.

Elective surgery is a common terminology, as explained above.

What do we already know about checklists like such and where does this study come into play:

Thank you for your comment, we have re-written the facts about current patient-driven checklist, authors believe the research gap is described clearly on page 2, lines 72-75:

"To-date, few checklists have been produced for patients to use in surgical care, and those that do exist are mostly tailored to specific surgical procedures rather than being generally applicable (7-10). Early evidence indicates that such patient-driven checklists might reduce hospital length of stay (LOS) and readmissions (7-9)." However, a reference on patient-driven checklist has been added to support the following statement on page 3, line 112-113:

"There is currently a knowledge gap regarding how a checklist tool like PASC may influence patients' involvement and perceived safety (10)."

'Post surgical' is a commonly used terminology within the surgical field. However, to clarify this we have removed 'post-' under the background section on page 3, line 113:

"Hence the aim of this study was to explore surgical patients' experiences of using PASC throughout their surgical pathway."

Further, we explain this more in the section 'Settings and participants' on page 4 lines 148-153:

"They were invited either before discharge from the hospitals or contacted after discharge via phone by ASH, KH, HWV, and RJT. If they accepted the invitation, an interview appointment was scheduled. No reminders were sent out. ASH, KH and HWV carried out the phone interviews, from a hospital office phone. No participants withdrew from this study. A total of 31 patients were interviewed approximately one to five weeks after surgery."

‘Surgical pathway’ commonly used terminology within our field. However, the authors have added a clarification on this term and have added an explanation in brackets to clarify this on page 3 lines 96-97

“The checklist is intended for use by patients, with the ultimate aim to empower surgical patients to actively participate in their own safety throughout their surgical pathway (from surgical preparation to recovery) (11, 12).”

Methods:

9. *Interviews were one-off, weren’t they? How could this study be a prospective one?*

   Thank you for your clarification, we have now removed prospective from the methods section on page 3, line 117

   “This was an exploratory qualitative study, based on in-depth individual patient interviews”

10. *You will need to provide some more detail about the Stepped Wedge Cluster Randomized Controlled Trial. What were participants supposed to do?*

    Thank you for this comment. We have now added more information about the trial in the methods section, on page 3 lines 118-

    “The study was part of a Stepped Wedge Cluster Randomized Controlled Trial (SWCRCT) (14), designed to investigate the impact of PASC on elective surgical complications, LOS in hospital, and mortality, as well as patients’ experiences with using PASC. Data in this trial were collected from November 2021 to February 2024. The elective surgical patients in the control group received care as usual, while the patients in the intervention group received PASC both electronically and by mail, one to eight weeks before surgery (14). In this part of the study, participants from the intervention arm were asked to share their experiences using PASC throughout their surgical pathway..”

11. *Remove any reference to COREQ in the main text, this is just for the review process.*

    The authors have not removed this due to editors comment.

12. ‘*Mentally and cognitively healthy’, how this was determined and by whom?*

    Thank you for your remark. We have now explained this in the text to make it clearer, under the setting and participants section page 4, lines 144-148:

“Patients who were eligible to participate were elective surgical patients ≥18 years, without cognitive impairment, fluent in Norwegian, and a part of the SWCRCT intervention arm utilizing the PASC intervention (14). The criterion for cognitive impairment was assessed by and in collaboration with the ward nurses in each cluster.”

13. *What does fluent in ‘intervention arm’ mean? You can only be fluent in a language.*

    Thank you for bringing this up. We see that this might be a bit unclear and have amended the text to clarify this, page 4, Line 144-147:

    “Patients who were eligible to participate were elective surgical patients ≥18 years, without cognitive impairment, fluent in Norwegian, and a part of the SWCRCT intervention arm utilizing the PASC intervention (14)”

14. *Lines 127-129 and Table 1 is Results rather than Methods. You wouldn’t know in advance how many would have participated. Also, did you carry on with recruitment until data saturation?*

    Thank you for your remark, we have now moved Table 1 to Results section, page 5 line 206.

    We mentioned saturation under data collection in our original manuscript. However, this has now been changed to ‘information power’ to ensure that we are in line with Braun and Clarks latest publication. The authors have brought in the Reflexive Thematic Analysis Reporting Guidelines (RTARG) guideline as a reference to ensure methodological coherence: *Braun V, Clarke V. Supporting best practice in reflexive thematic analysis reporting in Palliative Medicine: A review of published research and introduction to the Reflexive Thematic Analysis Reporting Guidelines (RTARG). Palliative Medicine. 2024;38(6):608-616. doi:10.1177/02692163241234800*

    See page page 5, line 170-173

    “Data information power was considered before the data collection. In addition, this was discussed and agreed upon by ASH, KH and HWV before analysis was carried out, as recommended by Braun & Clarke (18).”

15. *Was there any sort of recruitment material used? How were the interviews scheduled? Were any reminders sent?*

Thank you for your comment. The authors have moved this sentence from data collection to 'Settings and participants' based on comments from reviewer 1 and amended the text to ensure clarity. Please see page 4 lines 148-153:

"Patients enrolled in the trial were strategically sampled to participate in in-depth interviews post-discharge. They were invited either before discharge from the hospitals or contacted after discharge via phone by ASH, KH, HWV, and RJT. If they accepted the invitation, an interview appointment was scheduled. No reminders were sent out. ASH, KH and HWV carried out the phone interviews, from a hospital office phone."

16. *Lines132-150: Were the interviews in person or remote? Who carried out the interviews and what experience of qualitative research did they have?*

Thank you for this remark, we have added the following text under settings and participants, on page 5, line 153-154:

"ASH, KH and HWV carried out the phone interviews, from a hospital office phone. No participants withdrew from this study."

And under the section data analysis, on page 5, line199:
"All authors had extensive experiences with qualitative analyses"

17. *Were interesting topics arising during data collection explored in subsequent interviews?*

Thank you for this remark, authors have not addressed this comment based on the Analysis Reporting Guidelines (RTARG), see comment with reference above.

It is not a great practice having a 'rigid' topic guide in qualitative research.

Thank you for your comment. Apologies if this have been unclear, there is no ridge topic guide. We have used a semi-structured interview guide allowing participants to speak freely. However, to address our research question we needed to use the PASC items as a topic guide. This is also why Thematic Analysis with a semantic approach were chosen in this study. However, we have tried to clarify this issue by describing it in the text under Data collection, page 4, lines 162-167:

"Participants were initially asked to share openly their overall experience, including both positive and negative aspects, which resulted in detailed narratives of their experiences using PASC. Additionally, they were prompted to elaborate on specific PASC item topics such as surgical preparations, information needs, and discharge preparations. Finally,

they were asked whether PASC provided them with a sense of safety or not, and if they would consider using it again (supplement 2).”

The fact that there was no prior relationship between researchers and participants makes me questioning the honesty of participants’ views, how was rapport established between a bedridden and in discomfort (presumably) patient in a surgical ward and a researcher they never knew?

Thank you for bringing this up for discussion. We don’t see this as a problem, it would be impossible to have a prior relationship with the participants due to ethical considerations. However, we have tried to clarify the text around when and how the interviews were carried out both in our abstract on page 2, lines 48-49:

“An explorative qualitative study based on individual in-dept phone interviews with 31 elective surgical patients.”

and under the section settings and participants, page 4, line 148-153:

“Patients enrolled in the trial were strategically sampled to participate in in-depth interviews post-discharge. They were invited either before discharge from the hospitals or contacted after discharge via phone by ASH, KH, HWV, and RJT. If they accepted the invitation, an interview appointment was scheduled. No reminders were sent out. ASH, KH and HWV carried out the phone interviews, from a hospital office phone.”

18. *Lines 146-148: I don’t think there is a lot of appreciation as to what ‘data saturation’ mean. Data saturation usually has recruitment implications. You will need to state the precise model of saturation used and what this meant for recruitment.*

    Thank you for this comment. Please see our earlier comment to your question on data saturation. According to Thematic analyses; A practical guide by Brown and Clark page 28. “They state that such modelling of saturations must be avoided in this type of analysis.”

19. *Lines 148-250: I totally do not understand these sentences. Do you mean asking researchers to exclude their data? Otherwise they wouldn’t have been able to drop out, this was only a one-off interview (it is a black and white situation).*

    Thank you for your comment, and the opportunity to clarify the content. This was explained in the recruitment process, under data collection in the original manuscript. Patient were asked to participate prior to the interviews and none of them withdrew

from participating. To clarify this, we have moved a sentence from data collection to the setting and participants section to page 4 line152-154. Further, this is also a COREQ checklist requirement to report on.

"No reminders were sent out. ASH, KH and HWV carried out the phone interviews, from a hospital office phone. No participants withdrew from this study."

20. *Lines 153-159: I am sure I fully understand how exactly data analysis was done. How did you move from codes to categories, then to themes? Was there any framework used (e.g., Braun and Clarke) to guide the analysis process? What you present in Supplement 3 is not a coding tree. Quotations cannot be presented twice. Were there any disagreements amongst researchers when it came to data analysis? How these were resolved, if so?*
Thank you for your valuable remark. We referred to inductive thematic analyse, however we have rewritten some of the text to clarify this on page 5, line 178-

"Reflective thematic analyses with a semantic approach were used to analyze the data (16). In a semantic approach, themes are derived from the explicit or surface-level meanings within the data, with the analyst focusing solely on what participants have said, without seeking interpretations beyond the stated content (18, 19). This approach supported our research question and ensured both understanding and transparency within a multidisciplinary research team (medical doctors, general practitioner (GP), critical care nurses, nurse anesthetists, clinical nutritional experts, surgical ward mangers). Reflective thematic analysis proved to be a valuable method for KH, HWV, ASH, RJT and AS to gain an understanding of patients' experiences with using PASC, and to identify the patterns in the data that provided a broader understanding of how PASC influenced patient involvement in safety (16, 19). During the analysis the researchers KH, HWV, and ASH re-read all the interviews independently to get a sense of patients' experiences. Then, following a reflective discussion with HWV and ASH, KH and HWV revisited the transcript and composed a written reflection focusing on the individual stories. Next, KH and HWV coded the data with inductive descriptive codes with words or phrases on experiences or views with PASC. KH and HWV generated themes from the coding using mind mapping techniques. These themes and maps were discussed within several meetings with all authors. Based on these discussions, maps and the written reflections KH, HWV, RJT, AS and ASH finalized the analysis by conceptualizing the themes and reporting their content."

In addition, Braun and Clarks highlights in their latest publication 2024, that they recommend a simple citing and to avoid claiming to follow them.

We followed the steps of Braun and Clark which were mentioned in the text (se comment above). The Reflective thematic analysis moved from codes to themes as

described in their book: Braun V, Clarke V. Thematic analysis, A practical guide. SAGE publications Inc. 2022.

When it comes to supplement 3, we changed the title to ‘Coding Table, and replaced the quotation.

Results:

21. *Line 164: if themes are closely interconnected this means that analysis was not properly done, themes need to be mutually exclusive!*

    Thank you for your view on this, however we do not fully agree with you. According to Braun and Clarks papers they emphasize the importance of thematic interconnections, illustrated through models or maps that depict how themes relate to one another. *Braun V, Clarke V. Using thematic analysis in psychology. Qualitative Research in Psychology. 2006;3(2):77-101*. In addition, it is stated in their book: Thematic analyses a practical guide by Braun and Clarke, page 128, under the heading: Telling your Analytic story: “*Themes need to be fully developed in their own right and in relation to other themes.”* Further, on page 141, they state*: “although themes stand alone entities, there are typically moments of interconnection between them”.*

    In our study the themes are independent themes, but one theme leads to the other and vice versa, please see Figure 1. However, to minimise any misunderstandings on this, we have changed the text on page 6, line 208-210:

    “Three significant themes were generated through the analyses of the data, highlighting patients’ experiences with using PASC throughout their surgical pathway: PASC's influence on *patient awareness*, the stimulation of *patient actions,* and its perceived *utility value*.”

    And on page 7 line 215-219.

    “The three themes were based on multiple codes and were conceptually interconnected. The theme of patient awareness encompassed reflective elements that influenced patient actions, while these actions, in turn, reinforced awareness. The dynamic interaction between these two thematic constructs contributed to the third theme patients’ perceived utility of engaging with the PASC intervention (See Figure 1).”

22. *Lines 168-170: Remove these sentences, they say nothing to the reader.*
    Thank you for your comment, we have not removed these sentences, we have followed Brown and Clark’s Thematic analysis; a Practical guide were they recommend summing

up the themes before “telling the story”, as they call it in their book on page 130: *“often in reflective TA this takes form of a summary”.* Braun V, Clarke V. Thematic analysis, A practical guide. SAGE publications Inc. 2022.

23. *Lines 173-176: Apologies, this is not how qualitative research is being reported. Results present the themes, not the codes.*

    Again, thank you for your remark. We have removed the codes and focused on the themes. Please see the result section on pages 5-9.

24. *Apologies I am completely unable to follow the Results. It seems that very limited analysis has been performed and I am unable to comment any further. It seems that what is being currently presented is just a few scattered codes/concepts without any higher level of synthesis. Quotations are not being presented properly (e.g., after the quotation it is imperative to state Participant 1, 2, 3 etc., you can then state the ward if you want and all this should be presented in brackets). Quotations just repeat the narrative without providing any new data. The whole analysis should be repeated so that findings are presented succinctly and as per the standards of qualitative research.*
    We are sorry to read your view on this, however this analysis has a sematic approach, and recognize that we have not been very clear on this. We have therefore clarified in the ‘Data Analysis section’ on page 5 line 178-181:

    “Reflective thematic analyses with a semantic approach were used to analyze the data (16). In a semantic approach, themes are derived from the explicit or surface-level meanings within the data, with the analyst focusing solely on what participants have said, without seeking interpretations beyond the stated content (18, 19).”

    We have also adjusted the presentation of the quotations as you suggest under the result section on pages 5-9 and added a description on page 7, lines

    “Selected quotations are included represent personal insight and diversity within each theme. Participants citations are anonymized; we use “P” for participant and surgical specialty.”

    The comment on the quotations only repeats the narrative without providing any new data is understandable, however according to the RTARG guidelines mentioned and previously referred to; “Data extracts should convincingly and compellingly evidence the analytic claims”. Which is how the authors perceive to have used the quotations in this study.

Discussion:

25. *Once more apologies. Having not understood the Results, it is impossible to comment on the Discussion. At the moment arguments are all over the place and difficult to follow. Discussion sections should have the following structure: brief summary of findings within the first paragraph, comparison with existing literature in the next few paragraphs (each paragraph should be succinct and should flow well from the previous one), then a section about implications of the study before moving To Strengths and Limitations.*

    Thank you for your remark, we are sorry to read that this is how you experience this manuscript, we have followed your suggestion in the original manuscript with a brief summary of findings and then pulling inn literature. We find it hard to answer out this comment, based on reviewer 1 who has quiet opposite views on this manuscript. However, we have made some changes in the discussion based on reviewer 1comments and we hope this will clarify this section for you.

26. *Strengths and Limitations: arguments are not clear and I can only assume what the implied meaning is. E.g., 'another limitation could be that KH and ASH were the main developer of PASC and also interviewers', why is this a limitation? What sort of bias you are referring to? Please re-write this section in a clearer and more succinct way.*

    Thank you for your comment. It is stated in our text that this might favour biases, however, we have amended this part to make it more clear on page 11, lines 421-423:

    "Another limitation could be that KH and ASH were the main developers of PASC and performing interviews, which could lead to favor bias in relation to the data collection."

VERSION 2 - REVIEW

**Reviewer**	**2**
**Name**	**Karampatakis, Georgios Dimitrios**
**Affiliation**	**Queen Mary University of London, Wolfson Institute of Population Health**
**Date**	**05-Sep-2025**
**COI**	

I think the authors have addressed my comments to the best of their ability and manuscript is now ready to be published.
